# A Reflection on a Data Curation Journey

**DOI:** 10.1177/1556264615592846

**Published:** 2015-07

**Authors:** Lucia Lötter, Christa van Zyl

**Affiliations:** 1 Human Sciences Research Council, Pretoria, South Africa

**Keywords:** data sharing, data curation, institutional change management

## Abstract

This commentary is a reflection on experience of data preservation and sharing (i.e., data curation) practices developed in a South African research organization. The lessons learned from this journey have echoes in the findings and recommendations emerging from the present study in Low and Middle-Income Countries (LMIC) and may usefully contribute to more general reflection on the management of change in data practice.

The Human Sciences Research Council (HSRC) is a South African science organization that performs research aimed at improving understanding of social conditions. It receives public funding to support core activities but is dependent on contract or grant income to undertake major research projects. Several of its large-scale research projects involve nationally representative, cross-sectional repeat surveys dealing with attitudinal, behavioral, and health-related matters. Findings from these surveys are not only of interest to social scientists and the funders of research but also relevant to policy makers, students, journalists, and other users of social science data.

Since the early 2000s, when it published the findings from surveys such as the first South African National HIV Prevalence, Behavioural Risks and Mass Media Household Survey, the HSRC came under considerable pressure to make the data underpinning its research findings available to a wider audience of potential users. Actuarial scientists wanted to review and use the data to base forecasts on, and academicians from other institutions wanted to use the data to conduct their own analyses and make comparisons to feed into new publications.

Initially, there was a reluctance to share survey data. After all the hard work to obtain funding for what had been a risky survey to undertake; identifying key questions; developing and translating questionnaires; obtaining permissions; managing complicated fieldwork; capturing, cleaning, and analyzing the data; preparing the report; engaging with policy makers and the media, why should others be given access to the data and make this their resource from which to freely analyze, criticize, and gain publication credentials? Arguments against data sharing heard at this stage resonate with concerns identified in the studies appearing in this issue of the *Journal of Empirical Research on Human Research Ethics* (*JERHRE*).

In 2003, an international review panel recommended that the HSRC consider data management, including preservation and sharing thereof, as a critical part of its future role. In its cautious response to this recommendation, the HSRC (2003) highlighted the following concerns:

- There was no national policy around data sharing; hence, the question was raised why only one of several data-generating organizations in the country was required to share its research data with others.
- There was insufficient funding, infrastructure, and resources to make data publicly available and to serve the needs of potential users.
- There was a need to maintain confidentiality or anonymity of research participants, especially where this had been assured during informed consent processes.
- There were complexities around intellectual property rights, data ownership, and cost recovery, especially in a research entity that is dependent on contract or grant funding.

Other issues or concerns, identified during subsequent consultative workshops, included the following:

- There was some resistance to change, including perceived threats to competitive advantage, reluctance to change established work habits, and concerns about the additional burden of detailed recording of metadata.
- There was insufficient awareness of the value of data, and its potential re-use, combined with a lack of understanding of the risk of technology obsolescence, and its possible impact on future accessibility of research data.
- There were questions about infrastructure and resources, including what additional hardware and software would be needed to make data sets available and accessible to external users, and how would this be funded?
- There was discussion of potential risks associated with data management. There was a lack of shared standards for data management and a real risk that secondary or external users might find problems with the quality of some data sets.
- There were questions concerning new capabilities that would have to be developed, and the extra work that would have to be done, to make data available for others.

Despite these concerns, there were also drivers for change. Internal drivers included researcher needs. An in-house survey conducted in 2005 revealed that 94% of HSRC research staff regarded statistics/quantitative data as “very important” to their work, but only 41% regarded such information as easy to obtain. The fact that there was no formal platform for the sharing of HSRC quantitative and statistical data was regarded as a shortcoming, and the fact that data from important surveys were not always centrally deposited or managed was identified as a major risk for the organization.

External drivers included changes in the international research environment, where new initiatives to promote secondary access to data became more prevalent. This meant that there were increasing numbers of requests for data from external stakeholders and changes in the legislative environment.

## Early Adopters

By 2006, a core team of data management “champions” was ready to embark on a learning process. The team had a strong background in research, research data management, and systems development within the HSRC. Its members were keen to investigate ways in which data could be better managed, preserved, and made available for future use.

Their work was undertaken with very limited resources. It started with workshops involving senior researchers and research managers to raise awareness and do a needs analysis. This was augmented by international benchmarking and learning. As a first step to prepare for better data management, the existing project information system was extended to allow for the capturing of metadata of data sets. The team then started to work on data from one module of the South African Social Attitudes Survey undertaken in 2003 and developed an approach to clean, describe, and package the data set so that it could be made available on a platform that would be accessible to internal and external users alike. Steady progress was made with this pilot project.

Further workshops and awareness-raising road shows took place in 2007. A “framework for HSRC implementation” was presented and discussed. Challenges that had to be overcome at this stage included the development of “rules for access.” The aim was to formulate the rules in such a manner that external users would be able to access data as easily as possible, but that access would be managed and the confidentiality of individual participants, or even participants drawn from identifiable geographical areas, would be adequately protected. A dissemination interface linked to project information on the web was developed to prepare for the dissemination of pilot data by the end of 2007.

In February 2008, the HSRC co-hosted an international conference dealing with data curation—evidence of a small but growing community of data management practice in the country. This event developed into the annual African Conference for Digital Scholarship & Curation hosted by members of a community of practice called the Network of Data and Information Curation Communities (NeDICC; http://www.nedicc.com). The HSRC continues to participate in NeDICC activities.

## Accelerated Implementation

Toward the end of 2008, a new act was promulgated to confirm the purposes and objectives of the HSRC. One of the clauses of the act required of the HSRC to “. . . develop and make publicly available new data sets to underpin research, policy development and public discussion of the key issues of development, and to develop new and improved methodologies for use in their development” (Section 3(g). Human Sciences Research Council Act 17 of 2008).

Although no additional funds were made available to support this newly mandated objective, the HSRC chose to accept the challenge. With this new sense of urgency, more status was given to the team who had initiated work in the field of data curation. One of the objectives listed in the HSRC’s business plan for the 2008/09 financial year was to develop a long-term data curation, preservation and dissemination strategy for the HSRC.

Based on their earlier work and experience gained through international benchmarking, the data curation team could also develop policies and standard operating procedures (SOPs) for data curation. As had been their approach from the beginning, a consultative approach was followed to develop and periodically review these policies.

Engagement with the Research Ethics Committee (REC) of the HSRC ensured that plans for data preservation and sharing would, at least in principle, be built into research protocols from the outset. From 2011, all HSRC research protocols that were submitted for ethics review were required to be accompanied by a data preservation and sharing plan that would be reviewed by an expert in data curation. This required researchers to think more carefully about the kind of information they would provide to potential research participants about the envisaged use of research data and the kind of consent that would be required.

Managerial support was made even more evident in 2010, with the introduction of a new indicator of institutional performance that would be formally reported on annually—the number of research-generated data sets that had been preserved and, where appropriate, made available for future secondary re-use.

In the first year of implementation of this performance indicator, a target of one curated data set was established for each research program in the HSRC—in total, seven curated data sets for the organization. In subsequent years, the target for curated data sets was doubled and then trebled. From 2014, a limited cost-recovery model was introduced, and research programs were required to contribute a predetermined amount per curated data set to help cover the costs of activities directly associated with curation. At the time of writing, in March 2015, the HSRC has curated 87 data sets since the initial pilot project in 2007. The majority of these data sets are publicly available, but access to data is dependent on ethical requirements for protecting research participants, as well as on legal agreements with the owners, funders or in the case of data owned by the HSRC, the requirements of the depositors of the data. Data sharing is subject to an End User License agreement (http://curation.hsrc.ac.za/index.php?module=pagesetter&tid=125&;tpl=projects).

## Established Practice

In a period of approximately 10 years, the HSRC has experienced much change and growth in the area of data curation. By 2015, the following institutional practices were in place to support a data management culture:

Good governance, through approved policies and established practice
Processes to support good data management
  - A research management framework that highlights data management planning as a key component of research planning.
  - Research contracts that increasingly contain specific reference to the development of data sets and their subsequent ownership and management thereof.
Curation systems and processes
  - Systems include a metadata capturing interface based on the Data Documentation Initiative (DDI) standard (http://www.ddialliance.org), a file repository (for dissemination and preservation, respectively), and a dissemination interface linked to the HSRC’s website.
  - Processes include acquisition, preparing data and documents, producing metadata and preservation, and dissemination.
  - Guidance on preparation of data and data-related documents for curation (verification—including anonymization, describing data, publishing according to access parameters and preservation).
A dedicated team to provide support for data curation
  - Providing ongoing guidance to researchers, including training and other support to facilitate data deposit, preserving and sharing processes
  - Promoting the re-use of data
  - Building data curation capacity by expanding activities, and appointing and mentoring Research Data Curators
  - Interaction with the broader curation community
Support for the responsible conduct of research
  - In recognition of the belief that proper description and curation reduce the risk of scientific misconduct, curated data are carefully validated, checked, and annotated, and shared data are open for verification
  - A concerted effort is made to ensure proper acknowledgment of authorship and correct data citation, including assignment of Digital Object Identifiers (DOIs)
  - Attention to research ethics, including record keeping of consent provided, and special attention to anonymization and other methods to ensure non-disclosure of identities when data sets are combined or further analyzed
  - Access to data is managed through an end-user license
  - Working with the HSRC REC to review applications in terms of data-related matters

## Remaining Challenges and Opportunities

A remaining concern is how best to ensure appropriate recognition of the contribution of investigators and research teams who planned research, developed instruments, and collected and made available original data for further research analysis. If co-authorship of publications is not an option (and the debate may need to be re-opened at some stage), there is a need to insist on proper citation of data sets, so that the impact of good research surveys can be demonstrated. Continued funding for data collection and data management, undoubtedly the most cost- and time-consuming activities associated with original research, is dependent on on-going demonstration of the value thereof.

There is a lack of capacity to follow up on the secondary users of research data. Did they keep to the conditions of the end-user license agreement? Did they correctly cite the data that they used? Was it clear that they did a secondary analysis of data and were not responsible for the original data collection?

There is a need to promote secondary analysis of existing research data among postgraduate students and their supervisors. Training in research methods and the responsible conduct of research are seen as opportunities in this regard.

Researchers should be reminded to plan properly for secondary use. It is still not uncommon for researchers to limit themselves and potential other users of research data, by assuring potential research participants that the information gathered for the study will be strictly used for the purpose explained and for nothing else. Such a restrictive clause, of course, makes it much more difficult to allow re-use of research data unless explicit re-consent is obtained—and this is virtually impossible in most cases.

The HSRC’s data service has matured to such an extent that formal certification is the logical next step. Certification of the data service will further formalize the organization’s curation commitment and also provide owners, depositors, and users of data with assurances of trust that curation standards are adhered to.

There is a need for national policy and recognition for research data management, data curation in a national system of innovation. Research data should be considered as valuable research infrastructure, and the long-term preservation of research data should be prioritized as a national commitment.

## Discussion and Conclusion

It is hoped that the foregoing description of experiences in one specific institution may provide some encouragement and ideas for others to build on. To some extent, this journey of learning may also be viewed as a case study in change management.

As in many case studies of institutional change, the change that took place in the HSRC was mandated, and the institution had no choice but to comply. It is unlikely that change will take place in the same way in other organizations. In the HSRC, the “top-down” imperative was complemented and supported by strong and sustainable “bottom-up” processes. The introduction of data curation in the HSRC was planned, it started even before the legislation was promulgated and took place in an incremental and affordable manner. Most importantly, the HSRC case study has not been concluded as yet. There is still much to be learned, and to be done, also in collaboration with others.

Resistance or barriers to change often form part of and shape the change process. Various theories or models have been developed to describe the different phases of this process—for instance, the model based on the five stages of grief originally outlined by Elisabeth Kübler-Ross, namely, denial, anger, bargaining, depression, and acceptance (Kübler-Ross, 1969). These emotions and activities were evident among various role players, in various phases of the change process described above.

In his 1996 book *Leading Change*, John Kotter identified an eight-stage process of managing major change in an organization (Kotter, 1996). The steps are as follows:

1. Establishing a sense of urgency

There has to be a compelling reason to introduce the change. In this case, the (sustainable, “bottom-up”) compelling reason was the value of research data and the technologies that have become available to make these available for secondary use. Other institutions with valuable research data may also increasingly accept this compelling reason as their own. The sense of urgency was made very clear when legislation was changed in 2008 and performance measures introduced in 2010. In other institutions, external forces may include changes in international funding regimes and national legislation. A second compelling reason may be created by research partners from sponsor countries who are required to share data as a condition of their funding and collaboration.

2. Creating the guiding coalition

The HSRC was fortunate to have a strong, dedicated, and experienced team with skills spanning research, research data management, systems development, and information management, who took on the challenge of implementing change. In addition, the team found international and local curation communities to be very supportive and to have access to a huge knowledge base. The Internet is opening up new opportunities for collaboration and shared learning. It is conceivable that individuals or small teams from LMIC (Low and Middle-Income Countries) institutions may be able to build networks to start their learning journeys together.

3. Developing a vision and a strategy

One of the first things that the team did, under the auspices of the HSRC management, and in consultation with role players in the HSRC, was to develop a long-term vision for data curation in the HSRC. However, a long-term vision remains a dream if it is not broken up in smaller, achievable chunks that can be managed. Small steps are easier to take when the journey starts.

4. Communicating the change vision

There was a need for organizational buy-in to this vision. Even though the organization accepted that research data should be curated, resistance to a change in practice remained. Ongoing consultation and collaborative work were seen as key. Perhaps the most difficult part of the work related to commitment on an organizational level, on one hand, and on an individual level, on the other hand. Engagement with colleagues led to gradual co-ownership of ideas, not least of all because the ideas were improved by inputs from others.

5. Empowering broad-based action

Broad-based action was promoted with the introduction of performance measures across the organization. Even if all researchers did not participate in the process, they were at least aware of data curation activities and the importance thereof.

The implementing team, as well as researchers involved with data curation work, experienced this as a time-consuming and very exacting process. SOPs were developed and updated on an ongoing basis—one of the best contributions toward sustainability of the initiative. These SOPs will also serve as building blocks for future capacity building or expansion initiatives.

6. Generating short-term wins

The successful conclusion of the pilot project in 2007 was a significant milestone. The introduction of measurable performance targets ensured that each data set that had been curated would be recognized. On reflection of this particular case, the team leader remarked, “Resistance can be overcome to some extent by demonstrating success, but incentives are crucial. It was good to rather start small, and to keep track of achievements along the journey.”

7. Consolidating gains and producing more change

The lessons learned from initial implementation brought not only solutions but also new questions and new areas to explore. Implementation was described as “incremental and reiterative” and success as “not guaranteed to be sustainable.” This apparently pessimistic way of viewing change led to better planning for ongoing developments.

8. Anchoring new approaches in the institutional culture

Although great progress has been made, the challenges highlighted above show what more can be done to make data curation and support for secondary analysis of data a “way of life.”

In conclusion, reference should be made to more recent work of Kotter (2012) and Overbeck (2015). Overbeck made reference to “the power of baby steps” to help bring about change. Kotter has, since 1996, discovered the benefits of a “dual” operating system, working bottom–up and top–down. Successful, agile companies that move beyond the successful implementation of change (according to the eight stages described above) are those that also recognize that

1. Important changes are driven by many people
2. Voluntary participation is important—people should want to, rather than be told to, do something
3. Action should be driven by the head as well as the heart and should not only be aimed at achieving measurable objectives or numerical targets
4. More leadership is required, not only management
5. There should be a partnership between the “hierarchy” (management) and the “network”

To some extent, these lessons are relevant to the change process that the HSRC had embarked on.

## References

[bibr1-1556264615592846] Human Sciences Research Council. (2003). Human Sciences Research Council: Institutional review, 2003. Cape Town, South Africa: Human Sciences Research Council.

[bibr2-1556264615592846] Human Sciences Research Council. (2008). Business plan for the Human Sciences Research Council (HSRC) 2008/09. Pretoria, South Africa: Human Sciences Research Council.

[bibr3-1556264615592846] Kotter J. P. (1996). Leading change. Boston, MA: Harvard Business School Press.

[bibr4-1556264615592846] Kotter J. P. (2012, 11). Accelerate! Harvard Business Review, 90(11), 45-58. Retrieved from https://hbr.org/2012/11/accelerate 23155997

[bibr5-1556264615592846] Kübler-Ross E. (1969). On death and dying. New York, NY: Macmillan.

[bibr6-1556264615592846] Overbeck J. (2015, 2 26). You don’t have to be the boss to change how your company works. Harvard Business Review. Retrieved from https://hbr.org/2015/02/you-dont-have-to-be-the-boss-to-change-how-your-company-works

[bibr7-1556264615592846] South Africa. (2008, 9 30). Human Sciences Research Council Act, 17 of 2008. Government Gazette, 519(31470). Retrieved from http://www.gov.za/documents/human-sciences-research-council-act

